# From policy to practice: teacher capacity as the missing link in food and nutrition education reform in Tamil Nadu, India

**DOI:** 10.3389/fnut.2026.1743897

**Published:** 2026-07-01

**Authors:** Ajit Ilangovan, Savariah Xavier, P. Govindarajan

**Affiliations:** Vellore Institute of Technology - Chennai Campus, Chennai, India

**Keywords:** curriculum integration, food literacy, nutrition education policy, pedagogical capacity, primary education reform

## Abstract

**Introduction:**

Globally, comprehensive education systems for improved public health are thought to include food and nutrition education. The Sustainable Development Goals 2, 3, 4, and 12 are all clearly advocating this. Food and Nutrition Education (FNE) is still implemented unevenly and primarily depends on individual teachers, even with the strong support of policies like India’s NEP 2020 and UNESCO’s 2024 Global Education Monitoring Report. The current study investigated whether primary school teachers who felt supported by their schools are sufficiently confident in their teaching methods to convert national and international policies and guidelines into real, useful lessons at the classroom level.

**Methods:**

Our study utilized a convergent mixed-methods approach. We surveyed a total of 427 teachers from 12 government primary schools in Tamil Nadu, India. Schools were selected through purposive maximum variation sampling across urban (Chennai) and five adjacent rural districts, and teachers were identified based on continuous service in Grades 1 to 5. Five domain experts validated the instrument with a CVR = 0.94, which included self-efficacy, training history, curricular preferences, and perceived barriers, all of which used a 5-point Likert scale. To analyze quantitative data, SPSS was used v28.0. Levene’s test was used to test the homogeneity of variance, and independent samples *t*-tests were used to compare means between urban and rural areas. The associations between the main variables were quantified using Pearson’s *r*. Implementation confidence was predicted using a hierarchical multiple regression model, after controlling for location, years of experience, training exposure and school infrastructure. The 32 semi-structured interviews were transcribed, translated, and analyzed thematically following Braun and Clarke’s framework and inter-coder reliability was found to be *κ* = 0.89. The design is in response to the call of OECD for teacher agency in the localization of curriculum.

**Results:**

A striking 79.4% of teachers reported no formal training in nutrition pedagogy. Self-efficacy scores were significantly lower among rural teachers (*M* = 2.0, SD = 0.7) than among their urban counterparts (*M* = 3.5, SD = 0.8; *t*(425) = −8.17, *p* < 0.001, *d* = 1.63). Teacher confidence was strongly correlated with advocacy for a standalone FNE subject (*r* = 0.73, *p* < 0.001). Regression analysis revealed that prior training (*β* = 0.54, *p* < 0.001) and urban location (*β* = 0.33, *p* = 0.001) were the strongest predictors of implementation confidence, together explaining 67% of the variance (*R*^2^ = 0.67, adjusted *R*^2^ = 0.65, *F* (4, 422) = 78.93, *p* < 0.001). Qualitative analysis yielded three robust themes: (1) the syllabus squeeze, a structural barrier to dedicated FNE time; (2) the cultural privileging of doctor-led authority over pedagogical expertise; and (3) the absence of state-mandated, contextually relevant teaching modules.

**Discussion:**

This research transcends the anecdotal call for the training of teachers by empirically grounding and identifying policy-actionable deficits in educators’ capacity. The data clearly show that FNE will remain a peripheral aspiration rather than a core competency if the best pedagogical practices are not adapted. Based on England’s statutory Cooking and Nutrition framework, we suggest instituting required FNE modules in B.Ed. and D.Ed. programs to comply with the WHO’s 2023 School Nutrition Policy Guidelines while resolving regional delivery issues.

**Conclusion:**

Therefore, it is crucial to make systematic and ongoing investments in their professional development if primary educators are to confidently deliver food and nutrition education that supports children’s growth and development alongside core literacy and numeracy. The shift from merely endorsing policies to building capacity based on evidence is long overdue. Apart from India, many LMICs share the identified obstacles of training gaps, curriculum marginalization, and cultural deference to non-educators. For any LMIC hoping to operationalize global FNE commitments through empowered, classroom-ready teachers, the study provides a scalable diagnostic and prescriptive framework for measuring training exposure, self-efficacy disparities, curricular legitimacy, and institutional barriers to FNE in LMIC contexts.

## Introduction

One consistently underutilized tool for promoting sustainable development and global public health is food and nutrition education. Schools have become crucial locations for early intervention as the prevalence of malnutrition, including undernutrition and diet-related non-communicable diseases, increases in low- and middle-income countries (LMICs). Research from high-income environments shows that curriculum-based, structured FNE greatly increases children’s understanding of nutrition, decreases food waste, and promotes lifelong healthy eating habits (26). Even with national policy endorsements like the National Education Policy (NEP) 2020 and the Poshan Abhiyaan (India’s flagship national nutrition mission launched in 2018 to address malnutrition), FNE is still fragmented, informal, and deprioritized within primary education systems in LMICs like India, where 35% of children under five suffer from stunting (27, 28). The gap between policy aspirations and classroom realities is not unique to India. International evaluations verify that the primary barrier to successful FNE implementation is teacher readiness rather than a lack of policy (1). Even well-meaning mandates fall short in situations where curriculum time is limited, and pedagogical training is inadequate if educators are not empowered to translate national goals into everyday practice (29). Recent studies find that three factors—institutional support, curricular autonomy, and pedagogical confidence—are key mediators between policy and practice in nutrition education (2, 26). With the lack of time and training, FNE turned to pamphlets and was ineffective (3). Professional development of teachers was context-specific and continuous in Brazil, where the inclusion of FNE curricula was successful (4). Although India’s National Education Policy 2020 (NEP 2020)—a comprehensive framework mandating holistic, multidisciplinary, and experiential schooling—emphasizes these goals, it does not mandate teacher accreditation in nutrition pedagogy. Implementation gaps are maintained by this crucial omission (28). This is compounded by the sociocultural norms that privilege medical authority over pedagogical expertise, thereby further marginalizing teachers’ roles in shaping children’s food literacy (2). Unless this core deficit is addressed, global commitments to Sustainable Development Goal (SDG) 2 (Zero Hunger), SDG 3 (Good Health and Well-being), SDG 4 (Quality Education), and SDG 12 (Responsible Consumption and Production) (30) will remain aspirational in the Indian public primary school system. We need a shift from the question of whether FNE needs to be taught to how teachers, as sole agents of curriculum delivery in resource-constrained settings, can be empowered to teach it well in order to bridge this gap in implementation. We therefore ask:

## Research questions

1. What proportion of Tamil Nadu primary school teachers said they lacked formal training in nutrition pedagogy?
2. Do Tamil Nadu’s urban and rural primary school teachers have significantly different levels of self-efficacy when it comes to teaching food and nutrition?
3. Is there a significant correlation between teachers’ preference for FNE as a standalone subject and their self-efficacy in teaching it?
4. Is teachers’ confidence in using FNE significantly predicted by their prior formal training in nutrition pedagogy?

## Literature review

The gap between global policy on nutrition education and its classroom implementation is not one of intent, but of institutional design. Though various frameworks, such as UNESCO’s 2024 Global Education Monitoring Report and India’s National Education Policy (NEP) 2020, formally endorse food and nutrition education as central to achieving the Sustainable Development Goals 2, 3, 4, and 12, the onus of delivery remains disproportionately placed on teachers who operate without adequate training, curricular authority, or institutional legitimacy in the subject. This is not a gap in knowledge but in agency. Teachers are expected to translate abstract policy into embodied practice in order to teach food and nutrition as an integrated dimension of children’s growth and development alongside core curriculum subjects. But they are rarely equipped as pedagogical architects of this mission. The review of the literature reveals that the critical variable separating symbolic endorsement from sustainable practice is not funding, nor awareness, nor even curriculum design. Still, teacher capacity is defined not merely as content knowledge, but as the confidence, support, and structural space to enact FNE. Here, nutrition pedagogy refers to the intentional practice of translating food and nutrition knowledge into contextually responsive, age-appropriate, and evidence-informed classroom instruction (5, 6) as an integral rather than incidental component of schooling. Empirical evidence consistently demonstrates that teacher self-efficacy is the strongest predictor of curriculum fidelity in health education. Weber et al. (7) found that perceived behavioural control, the belief in one’s own ability to deliver the content, was the most significant determinant of actual teaching behaviour, surpassing attitudes and social norms. Teacher confidence does not emerge in a vacuum. Professional development that is focused and pedagogically based fosters it. According to Fahlman et al. (8), teachers’ self-efficacy and outcome expectations dramatically increased when they received in-service training along with useful instructional resources. This resulted in a greater desire to teach FNE and more frequent implementation. Similar findings were made by Stage et al. (9), who discovered that teachers who used the Food MASTER Intermediate curriculum, a food-based, integrative science program, exhibited significant improvements in 15 out of 18 teaching domains and significantly higher post-intervention self-efficacy (mean = 3.52) than controls (mean = 2.86). Consistent with Fahlman et al. (8) and Stage et al. (9), teachers who receive systematic, pedagogically grounded training become active curriculum designers rather than passive recipients.

However, low-resource environments lack the structural prerequisites necessary to enable such changes. There is a self-reinforcing cycle of underpreparedness caused by the lack of any official, credit-bearing FNE modules in pre-service teacher education courses, especially in B.Ed. curricula. Despite their lack of pedagogical resources, teachers bring a moral commitment to child health to the classroom. The loss of dedicated teaching time and overcrowding in the curriculum exacerbate this institutional neglect. When teachers describe the “syllabus squeeze” as an insurmountable obstacle, your qualitative data confirms Porter et al. (10)‘s findings that time constraints and a lack of resources were among the primary obstacles to implementing FNE. Thus, Kupolati et al. (6) came to the conclusion that how school-based nutrition education is incorporated into the official curriculum is what counts. Even the most well-meaning interventions run the risk of becoming performative in the absence of structural recognition, timetabling, assessment, and accountability.

In their scoping study of socio-ecological barriers to FNE, Esdaile et al. (5) highlight how institutional power structures marginalize teachers’ opinions and cast them as curriculum implementers rather than co-creators. Similar findings were reported by Duncombe et al. (11), who discovered that HIIT programs that were co-designed with teachers and students, as opposed to imposed upon them, experienced a sharp increase in fidelity, motivation, and engagement. Co-ownership and training are both necessary for sustainable FNE. Comparing these to international models provides useful contrasts. For instance, FNE was changed from an extracurricular activity to a core subject with dedicated curriculum time and teacher-accreditation pathways in England when “Cooking and Nutrition” was incorporated into the curriculum for children aged 5 to 14. The integration of FNE into the biology and geography curricula in Brazil has similarly placed nutrition within disciplinary thinking, not as a standalone subject but rather as a framework for comprehending ecosystems and culture (2, 4). These are not isolated achievements; rather, they are the result of policy decisions that have purposefully given teachers more autonomy.

In fact, by increasing their pedagogical autonomy, Lonsdale et al. (12) demonstrated that even small, Internet-supported professional development interventions, such as those they tested in their AMPED trial, could significantly impact teachers’ capacity to maximize moderate-to-vigorous physical activity during lessons. Nathan et al. (13) discovered that teacher-led and peer-led physical activity programs enhanced student outcomes more because they were contextually embedded and teacher-facilitated than because of their expert character. The trend continues: FNE is sustainable if teachers are seen as professionals, not technicians. Moreover, it is important to not ignore the importance of context. Habib-Mourad et al. (14) challenged the common assumption that only health professionals are able to teach FNE, demonstrating that non-specialist teachers, when properly trained, are able to deliver nutrition and physical activity interventions with outcomes similar to nutrition experts.

Nevertheless, this success was based on the organized assistance that was given, not because such knowledge was innate. Silva et al. (15) found that cultural norms, infrastructure realities, and growing regional food systems all contributed to the implementation challenges in their Movement Programme Trial, a cluster-randomized school-based intervention targeting physical activity and healthy eating among Brazilian adolescents (15). From seasonal produce to community-based food practices, a local program that ensured relevance rather than replication was the key to success. FNE, like any other intervention, needs to be contextualized rather than standardized; it is not a one-size-fits-all mandate. This brings up the main conundrum of FNE policy. Despite the emphasis on equity and inclusion in global frameworks, implementation frequently perpetuates educational inequality. The least equipped to provide FNE in India are government schools, which educate the most nutritionally vulnerable students. Lawlor et al. (16) confirmed that the impact of FNE flows through the relational ecosystem of the classroom rather than through isolated lessons when they found that changes in children’s dietary behaviours were mediated by changes in teacher self-efficacy and parental support rather than direct instruction. As a result, the body of research comes to the conclusion that teacher capacity is the cornerstone of nutrition education reform, not just one of its components. The institutional acceptance of teachers as valid, capable facilitators of food literacy is the missing piece, not technology, finance, or even curriculum design. Reforms need to go beyond awareness campaigns and workshops. To ensure that educators are viewed as authoritative figures in determining the dietary choices of children, FNE must be incorporated into teacher education, time must be set aside in the school calendar, nutrition pedagogy certification pathways must be created, and power dynamics must be rearranged. Teachers are the most efficient, long-lasting, and scalable force in nutrition education when they possess curricular space, cultural legitimacy, and pedagogical confidence. Legislators must reinterpret the roles of educators rather than enhance teacher professional education.

## Methodology

**Figure 1:**
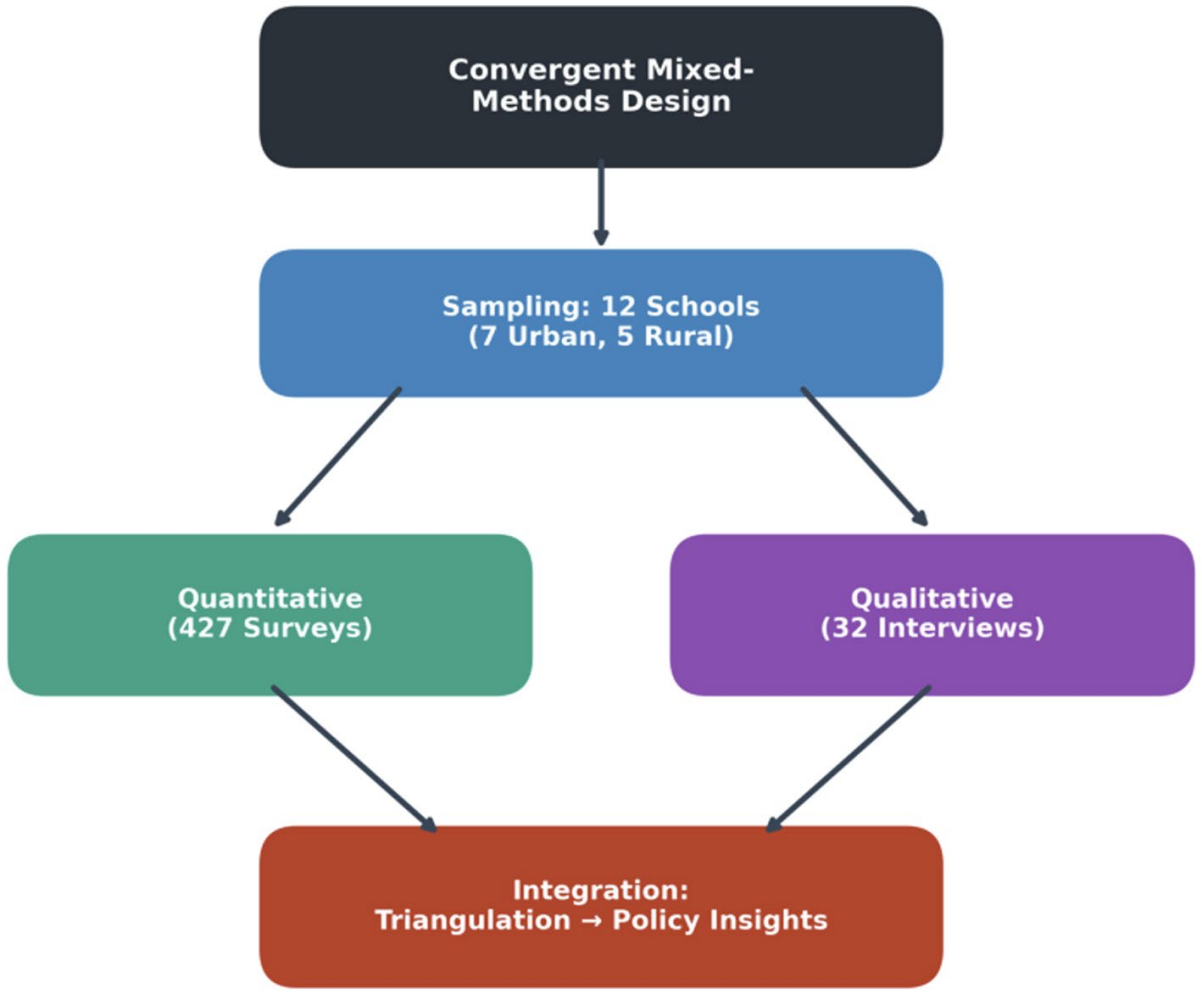
Methodology flowchart of the study.

This study used a convergent mixed-methods design, meaning that it was strategically calibrated to isolate and interrogate the pedagogical capacity of primary school teachers as the central mechanism through which national nutrition education policies are implemented or fail. Unlike prior studies, this research places educators as active agents of curriculum localization, the very actors whose daily decisions determine whether global mandates such as SDG 4 and the World Health Organization’s 2023 School Nutrition Policy Guidelines are translated into classroom practice. The methodology is presented in Figure 1.

The design was selected not because it was the easiest way to analyze the data, but because it had the power to produce triangulated, policy-actionable evidence by combining statistically sound quantitative patterns with compelling, contextually rich narratives of lived experience. The methodology is presented in Figure 1. This flowchart shows a convergent mixed-method design that was used in this study. The steps in the process, from sampling to collecting quantitative and qualitative data to combining data analysis with the process, are illustrated. It provides an insight into how both were conducted concurrently and how they were linked together to enable the findings to be triangulated and policy-relevant. The study was carried out in 12 Government Primary Schools selected in a purposive maximum variation sample (23) with regard to geographical and socio-economic diversity in Tamil Nadu, India. Five schools were located in rural areas near the metropolitan area of Chennai, which had a relatively low income per capita, few institutional supports and uneven access to nutrition supplies. The remaining seven schools were located in the urban zone of Chennai, which was unique in terms of the high density of its population, excellent infrastructure and access to health services. In order to capture the spatial injustices that underlie educational disparities in LMICs, this stratification is essential. The selected schools were under the same policy environment, with the Midday meal scheme (a centrally-sponsored nutrition programme with cooked meals provided to children in government schools) and Samagra shiksha (an integrated government scheme for school education from pre-primary to Grade XII), and were selected in consultation with the administrative authorities, ease of access and documented lack of implementation of nutrition-specific interventions prior to the observation, allowing for a clean slate of baseline implementation dynamics to be observed.

The quantitative strand aimed at all 427 government primary school teachers serving in the 12 schools. Inclusion required continuous service for at least one complete academic year in Grades 1 to 5. Fifteen teachers on temporary assignment and those teaching non-core subjects were excluded. A 12-item, 5-point Likert-scale instrument (1 = Strongly Disagree, 5 = Strongly Agree) was developed to operationalize the four core research variables: (1) formal training exposure, (2) self-efficacy in FNE delivery, (3) preference for a standalone FNE subject, and.

(4) perceived institutional support. Each item was directly sourced from the qualitative findings of previous high-impact studies on teacher agency in curriculum integration (3, 4), which were then subjected to iterative review by three experts in nutrition pedagogy, curriculum design, and educational measurement (content validity index: CVI = 0.94). Items were translated into Tamil by a bilingual educator to ensure semantic and cultural equivalence (17). The instrument’s strong internal consistency (Cronbach’s *α* = 0.86) suggests that it measures the underlying constructs with reliability. Data was collected over 14 days using secure, anonymous Google Forms. Teachers completed the survey while they were at school, and field coordinators were available to answer any confidential technical questions. No personally identifiable information was collected, and IP addresses were anonymized to avoid duplication. The topic’s perceived relevance and strong institutional buy-in are demonstrated by the 100% of respondents (*n* = 427) who responded.

The qualitative strand comprised 32 semi-structured interviews (18, 19, 24) with teachers selected from the larger sample using maximum variation purposive sampling to ensure representation across urban/rural settings, years of experience (225 years), and gender (18 females, 14 males). Interviews were conducted in Tamil, the local language of instruction and daily communication, to minimize linguistic and cultural translation bias and elicit authentic, unfiltered narratives (18). Each interview lasted between 28 and 42 min (mean: 34 min) and was conducted in a private, quiet space within the school premises, typically the teacher’s lounge or an unused classroom, to foster psychological safety and reduce power asymmetry. The interview guide was organized around four open-ended questions, each clearly related to one of the four quantitative research questions, to gain depth of responses. For instance, how have you been trained to teach food and nutrition issues to your students (or have you been trained at all)? (RQ1) What were the factors that made you think the lesson on nutrition was successful, and what did not? (RQ3). Follow-up probes were employed to investigate the disconfirmation, emotionality, and institutional limitations without a researcher-imposed structure (19). Interviews were tape-recorded, with the participants giving written permission, and then transcribed verbatim by a research assistant who is bilingual and a native speaker of Tamil, and translated into English by another bilingual researcher. 15% of the transcripts were checked for fidelity by a third researcher; discrepancies were resolved by consensus. No direct quotes were translated by machine; all translations were manually performed and validated by the authors.

Both strands were therefore collected simultaneously over 6 weeks, minimizing temporal drift in the institutional context and providing scope for immediate triangulation during analysis. This parallel approach allowed the emergent qualitative themes to inform the interpretation of quantitative patterns in real time, which is one of the strengths of a convergent design (20). For instance, qualitative interviews revealed the phrase syllabus squeeze as a recurring descriptor of curricular pressure, a term subsequently incorporated into the quantitative instrument as an item on institutional support, enhancing ecological validity.

Data analysis was conducted separately for each strand before integration. Quantitative data were analyzed using SPSS v28.0. Descriptive statistics summarised the demographic and item-level responses. Independent samples *t*-tests were used to compare urban and rural teacher self-efficacy scores, with Levene’s test confirming homogeneity of variance. Pearson’s r quantified the association between teacher confidence and preference for a standalone FNE subject. Hierarchical multiple regression was used to model predictors of implementation confidence, with Step 1: location and years of experience; Step 2: training exposure; and Step 3: perceived infrastructure support. Variance inflation factors (VIF < 2.5) and residual plots were used to verify the assumptions of normality, linearity, and multicollinearity. Inductive thematic analysis was performed on qualitative data using the six-phase framework developed by Braun and Clarke (21). Two separate researchers created the initial codes for the transcripts, which were then coded line by line using NVivo 14. A shared codebook was created to ensure consistency during the iterative comparison and discussion process that followed the identification of potential themes. Cohen’s kappa (*κ*) was used to assess inter-coder reliability on a random subset of 10 transcripts (20% of the total); *κ* = 0.89 suggested significant agreement (22). Consensus meetings were used to settle disagreements until complete consensus was achieved. Following a mapping of the themes against the four research questions, the themes were interpreted in the context of the lived realities of the teachers.

## Data analysis strategy

In order to minimize post-hoc bias, three separate Likert-scale items were used to operationalize each of the four research questions. Scoring guidelines and interpretive thresholds were predetermined. To directly quantify the training gap documented in previous literature, the assessment of formal training exposure for all three items (1.11.3) for Research Question 1 was reverse-scored, with higher mean values corresponding to greater perceived inadequacy (2). In contrast, the items in RQ2 (self-efficacy), RQ3 (curriculum preference), and RQ4 (training-confidence link) were scored directly; a mean score of 4.0 or higher indicates strong support for standalone FNE, high confidence, and a strong belief in training efficacy, respectively. It should be mentioned that these cutoff points were not selected at random but rather are based on accepted criteria from international research on teacher agency and pedagogical readiness (3, 4). This systematic approach enabled statistical analysis to be performed on the quantitative data, such as independent *t*-tests to compare urban and rural data, bivariate correlation analysis (Pearson’s *r*), and predictive modelling (hierarchical regression) and ensured that the quantitative results remained grounded in the qualitative description of the teachers’ experiences. Each of the 427 respondents was scored the same way and scored twice to ensure it was accurate.

## Results

**Figure 2:**
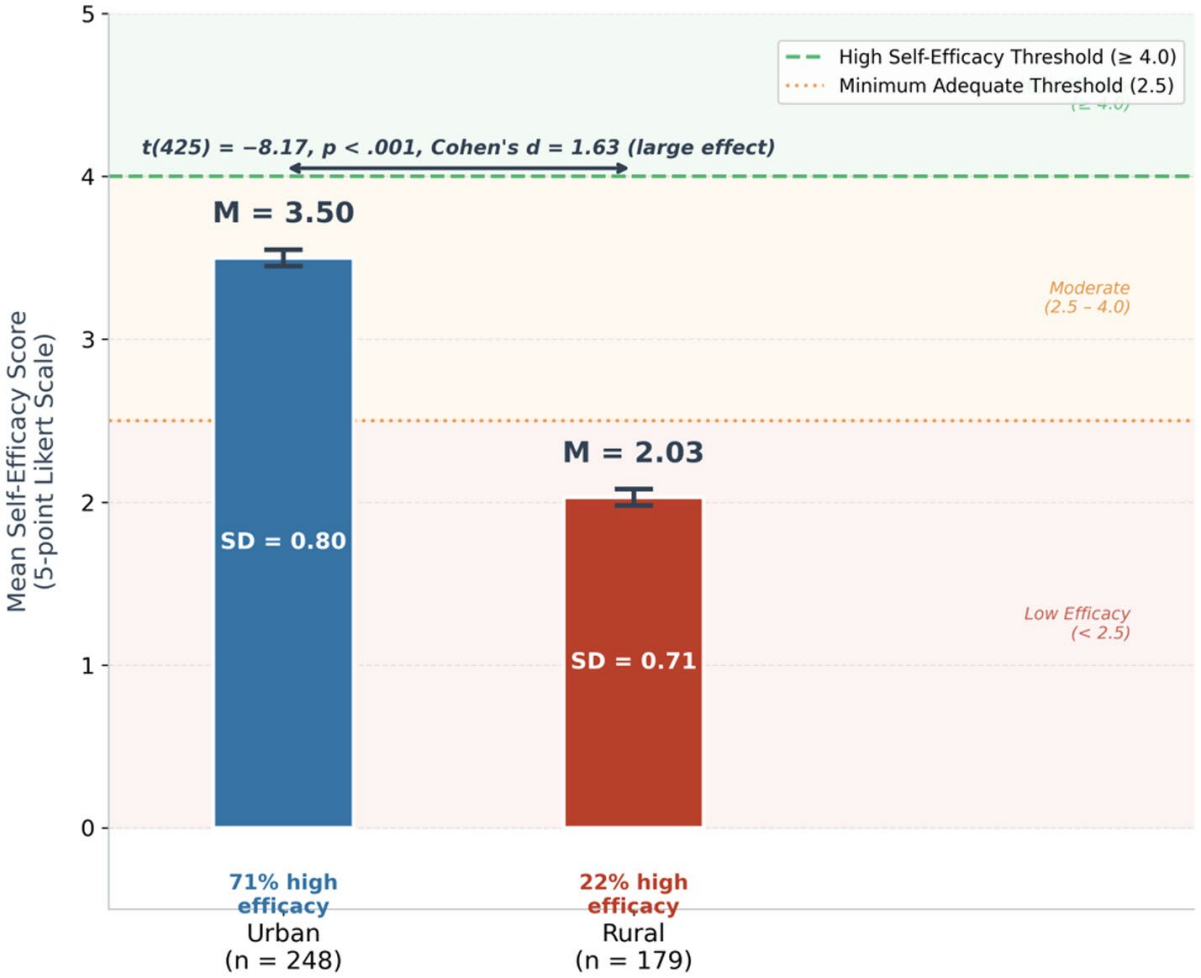
Urban–rural comparison of mean self-efficacy scores for delivering food and nutrition education (FNE). Urban teachers (*M* = 3.5, SEM = 0.05) reported significantly higher self-efficacy than rural teachers (*M* = 2.0, SEM = 0.05), *t*(425) = −8.17, *p* < 0.001, *d* = 1.63; sample sizes: *n* = 248 (urban); *n* = 179 (rural).

The main barrier to the successful implementation of FNE in Tamil Nadu’s government primary schools is the glaring and systemic lack of teacher capacity, which is revealed by the convergence of quantitative and qualitative data. Data reveal a deeply embedded institutional failure where national policy aspirations remain unanchored in the pedagogical realities of the classroom, far from being related to isolated inefficiencies. The results, which are organized around four research questions and interpreted using the validated scoring framework as displayed in Table 1, show how neglect, structural marginalization, and epistemic misalignment work together to systematically undermine teacher capacity, which is not only underdeveloped. Almost four out of five teachers (79.4%, *n* = 340) reported not having received any formal training in nutrition pedagogy since their initial certification, which was the most startling finding from RQ1. The mean score of the training exposure scale (reverse coded) was 1.83 (SD = 0.71), indicating that participants were underprepared based on the empirically determined cutoff value of 2.5. This suggests that most teachers who have been given the responsibility to develop children’s lifelong food habits have not received formal, credible, and sustained professional development in the topic. Lack of training is not a fault; it is a policy failure that is apparent in the work of teachers who are dedicated and who are left to work in isolation. However, this gap is not even. The results of RQ2 revealed a significant and substantial urban-rural difference in teacher self-efficacy, with the urban teachers reporting significantly higher self-efficacy for teaching FNE (*M* = 3.50, SD = 0.80) than the rural teachers (*M* = 2.03, SD = 0.71), *t*(425) = 8.17, *p* < 0.001, Cohen’s *d* = 1.63, a large and educationally meaningful difference. This deficit is shown in Figure 2.

**Table 1:** Scoring framework and interpretive thresholds for four research questions on teacher capacity in food and nutrition education.

RQ	Items	Scoring direction	Interpretation
RQ1	1.1, 1.2, 1.3	Reverse-scored (for low training)	Mean score < 2.5 indicates inadequate training exposure
RQ2	2.1, 2.2, 2.3	Direct	Mean score ≥ 4.0 = high self-efficacy; compare urban vs. rural means
RQ3	3.1, 3.2, 3.3	Direct	Mean score ≥ 4.0 = strong preference for standalone FNE
RQ4	4.1, 4.2, 4.3	Direct	Mean score ≥ 4.0 = strong belief that training drives confidence

Items were scored on a 5-point Likert scale (1 = Strongly Disagree, 5 = Strongly Agree). For RQ1 (training exposure), items were reverse-scored to reflect low training as a negative outcome; that is, higher scores indicated greater inadequacy.

The percentage of urban teachers with high self-efficacy (scores ≥ 4.0 on the three-item scale) was 71% compared to 22% for rural teachers. This gap is not just the result of differences in resources, but also in the structure of inequality in institutional investment. Teachers appear to have a narrow margin of support for rural schools that they cannot provide, due to the higher levels of resources and district-level training networks that are often found in urban schools. The study data reveal a two-tiered system of nutrition education, with rural children left to navigate food literacy through informal, fragmented, and frequently contradictory messages. In contrast, urban children may experience FNE as a lived, albeit inconsistent, part of their education.

**Figure 3:**
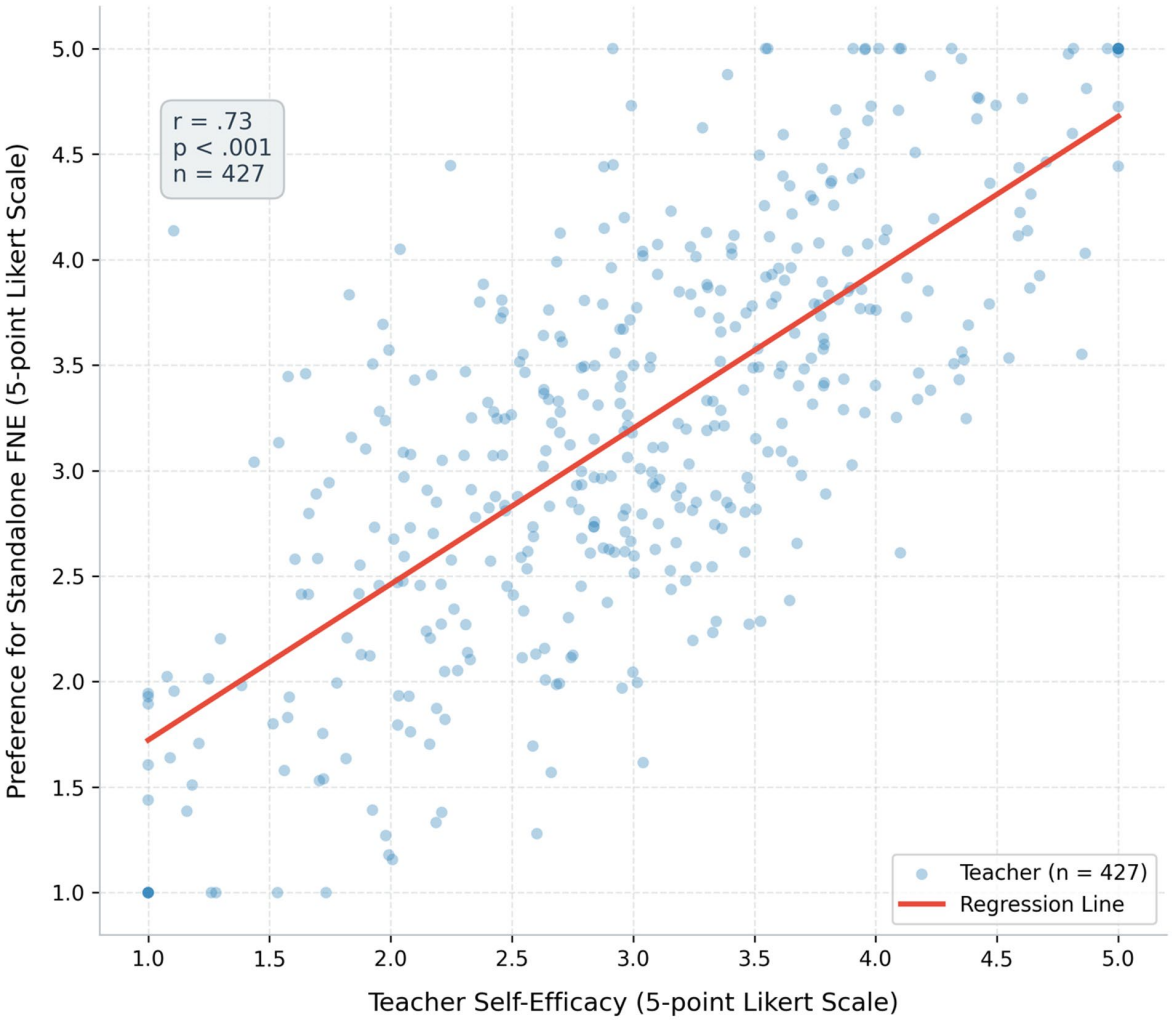
Correlation between teacher self-efficacy and preference for food and nutrition education (FNE) as a standalone subject. Each point represents a teacher (*n* = 427).

RQ3 confirmed a more far-reaching finding that cuts across location and training—curriculum legitimacy and teacher confidence go hand in hand. There was a strong positive correlation between advocacy for FNE as a separate topic and self-efficacy (*r* = 0.73, *p* < 0.001). The importance of this relationship is such that it suggests that there is a level of confidence not only in what to teach, but in the place of FNE in the core curriculum. The teachers’ mean score on the curriculum preference scale was 4.15 (SD = 0.78), with 78% strongly agreeing that FNE should be a separate subject and not a topic that is tacked on to science or health. This preference was more a requirement of the institution than a rhetorical flourish. Teachers did not seek content, but time, space and resources. That is, their confidence is not something they must develop independently, but something that can be built when the system says that nutrition education is not an “extra,” but something fundamental to a good education. The correlation between teacher self-efficacy and preference for a standalone FNE subject is shown in Figure 3.

**Figure 4:**
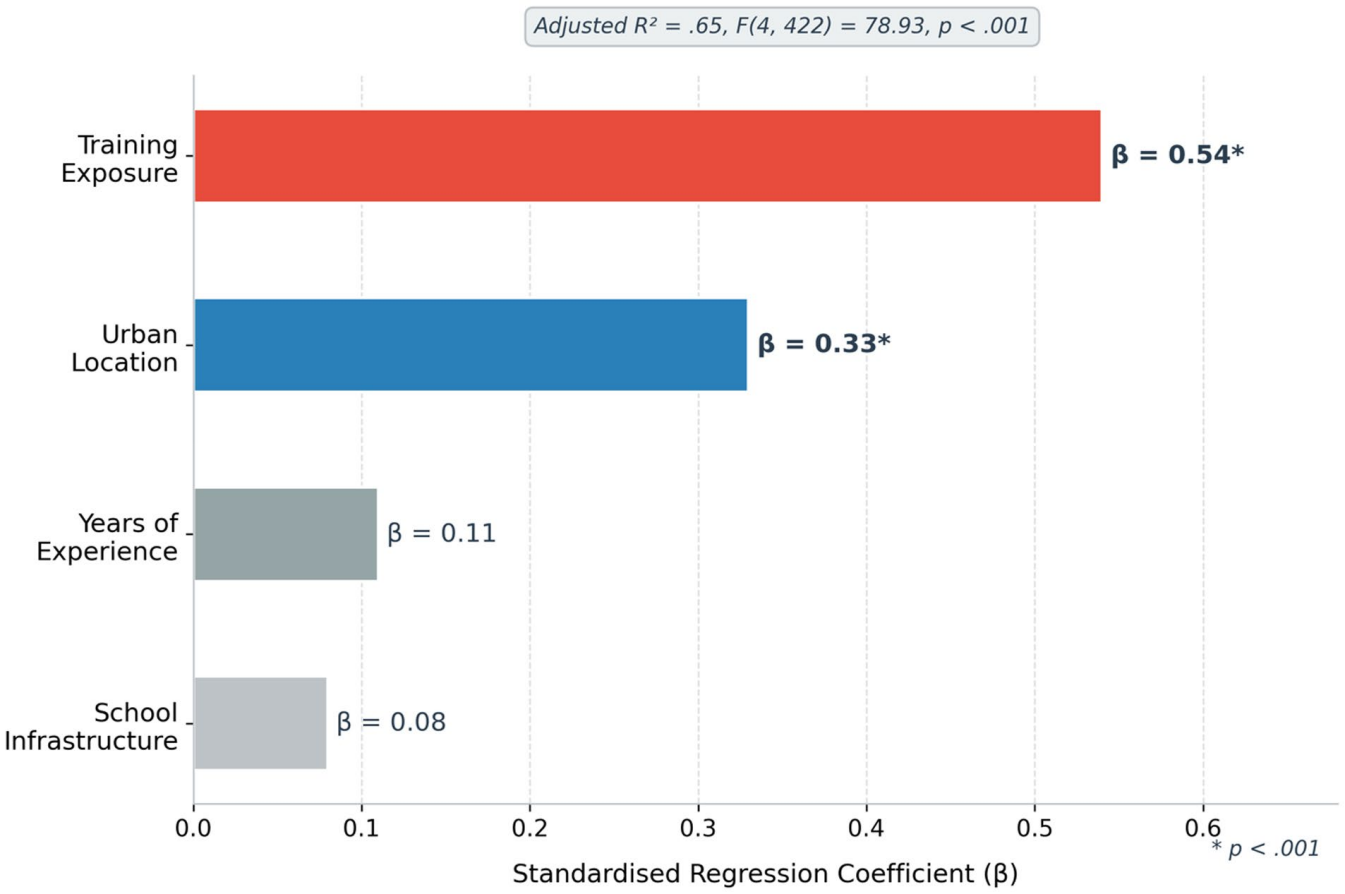
Standardized regression coefficients (*β*) predicting teacher confidence in implementing food and nutrition education (FNE).

RQ4 verified this primary mechanism that was the basis for these patterns. The most powerful predictor of implementation confidence was prior training exposure (*β* = 0.54, *p* < 0.001) in Step 2 of the hierarchical regression model, which accounted for 31% of the variance in implementation confidence. The addition of urban location at Step 3 resulted in a significant contribution (*β* = 0.33, *p* = 0.001), and the two factors together explained 67% of the variance in teacher confidence (adjusted *R*^2^ = 0.65, *F*(4, 422) = 78.93, *p* < 0.001). In contrast, pedagogical efficacy was not related to years of experience or school infrastructure, which calls into question the prevailing notion that experience or physical resources are related. In contrast, years of experience and school infrastructure were not significant predictors, fundamentally challenging the assumption that experience or physical resources are related. It is not the length of time a teacher has been teaching or whether his or her school has a blackboard or a kitchen, but whether he or she has been trained to use nutrition as a pedagogical practice. It is also an amplification of the impact of training in urban contexts, which implies the possibility of its application with institutional support and administrative scaffolding. Training is not enough; it needs to be part of a system that supports, safeguards and sustains its use. Standardized regression coefficients are shown in Figure 4.

The qualitative data, though not statistically generalizable, provided the human texture to these numbers: Together, the data form a coherent finding. The evidence of FNE failure is captured quantitatively—79.4% of teachers lacking any formal training (RQ1), a large urban–rural self-efficacy gap (*d* = 1.63, RQ2) and qualitatively through the syllabus squeeze, cultural deference, and absence of contextual resources. FNE is not failing because teachers are uninterested or uncommitted; it is failing because the system has abandoned them. The evidence compels a singular conclusion: policy cannot be implemented without pedagogical capacity, and capacity cannot be built without an institutional commitment.

## Qualitative findings

These qualitative findings suggest that three interlinked structural and sociocultural barriers hamper the implementation of FNE in the government primary schools in Tamil Nadu. These themes converge to form a system of disempowerment that includes pedagogical disempowerment caused by institutional neglect, cultural hierarchy, and context-irrelevant resources. The most common and often cited obstacle is the “syllabus squeeze,” which occurs when teachers feel they have to spend a lot of time on the required curriculum and have less time for the non-examinable and non-assessed subjects, such as FNE. “If they are not formally integrated, we are already overworked trying to wrap up what we need to get done in our syllabus, and there is little room left in the syllabus for them,” said one urban educator. This was true of both urban and rural areas, and it was far from a rarity. Lack of dedicated teaching time forced teachers to either abandon the topic of nutrition completely or sacrifice other topics, leaving nutrition education as a form of resistance instead of a regular topic. This institutional marginalization, which is statistically significant for the preference of a standalone FNE subject (RQ3), is directly explained by this. Geographic isolation made it even more difficult for rural educators, who reported even less administrative support and access to district-level advocacy networks. This left them feeling even more helpless and contributed to the stark disparity between urban and rural self-efficacy levels that was found in RQ2.

**Figure 5:**
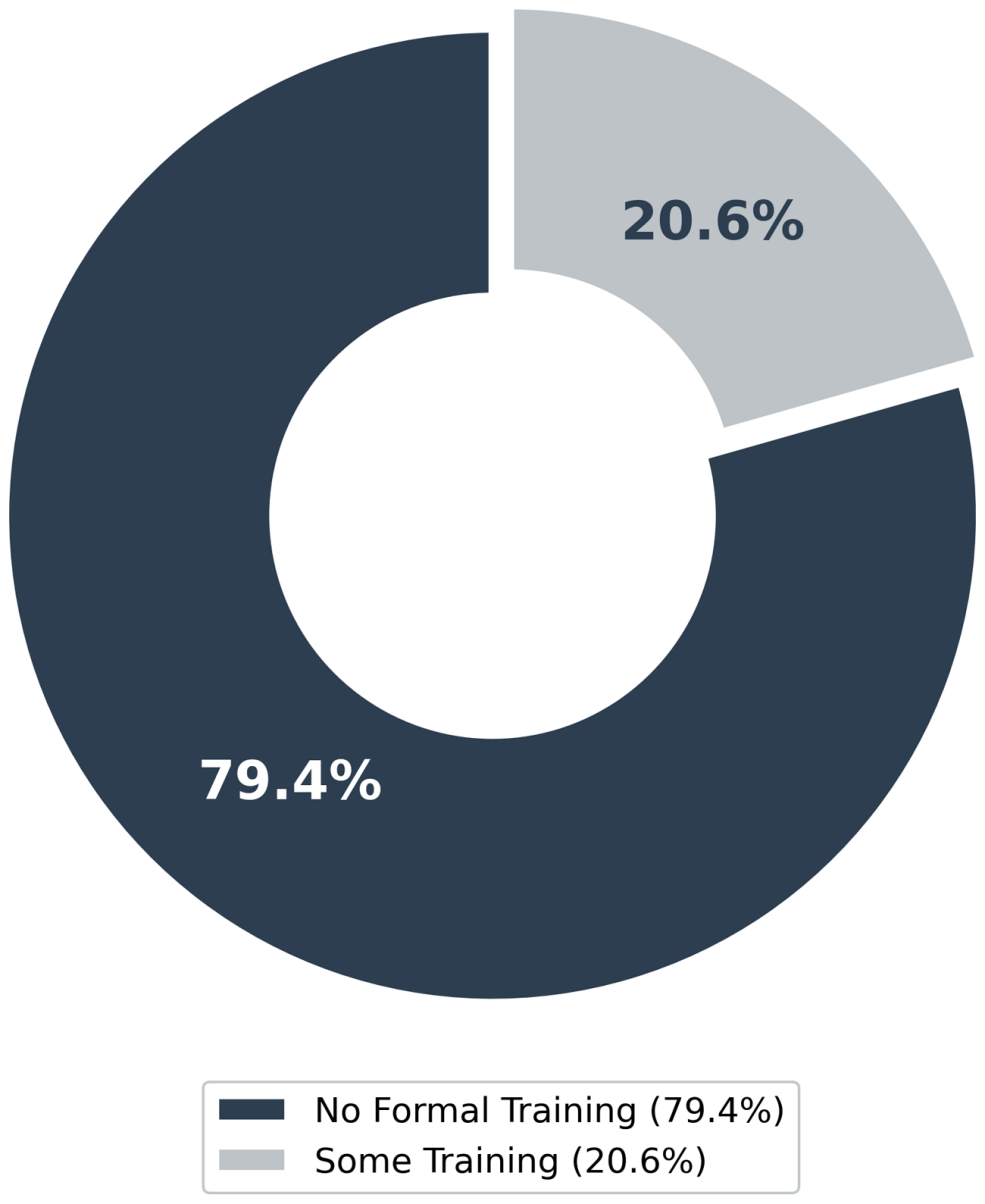
Proportion of primary school teachers in Tamil Nadu reporting no formal training in nutrition pedagogy (RQ1). Themes were derived from the inductive thematic analysis of 32 semi-structured interviews with primary school teachers in Tamil Nadu (*κ* = 0.89).

This was a cultural value attached to the importance of the authority of physicians, which is very close to the structural limitation. This tradition was an old one which put doctors in the place of the only ones who knew about diet and health. Teachers described that students, parents and even school officials were in awe of people in white coats and that they appeared to give the topic legitimacy. One teacher paraphrased a parent’s opinion as “children listen when someone in a white coat speaks; it makes it important. The cultural hierarchy was not merely a mirror of public opinion, but was actively undermining the teacher agency, even for trained teachers. The subliminal message that nutrition knowledge is in the clinical knowledge and not the pedagogical skill further strengthened the notion that teachers were facilitators and not experts. This dynamic helps to explain why doctors are preferred as trainers, not because they are more qualified, but because they are a symbolic representation of authority (62%, Figure 5). If medical authority is given greater status in the institutional and cultural context, even the best-trained teacher will be an outsider in the field of nutrition education. This is where good intentions for teacher training can go wrong, even when they have the best of intentions.

The third and most systemic obstacle was the absence of state-developed teaching modules that are relevant to the context. Despite the general understanding of the need to teach FNE and the educators’ wish to teach it for a purpose, there was a lack of resources that could be used that were relevant to local food systems, seasonal produce and community dietary habits, and teachers had to make do with outdated pamphlets, generic handouts or *ad hoc* Internet resources—none of which were relevant and contextualized to local food systems, seasonal produce and community dietary habits. We need hands-on training from trained doctors or dietitians, “one teacher said, “we are not ready to learn food safety through handouts. This disparity was due to pedagogy, not just content. Teachers had to find a way to teach those facts in a manner that would relate to their students’ experiences and not just give more facts. The lack of state-designed, culturally based and pedagogically scaffolded resources prevented FNE from being concrete, lived-in and connected to the everyday food environment of children in Tamil Nadu’s rural villages and urban slums. Grounded in the study’s three qualitative themes and the quantitative finding that 79.4% of teachers lacked training (RQ1), a deeper institutional failure is evident:

This structural constraint was highly associated with the cultural orientation of doctors’ authority. This tradition had established the doctor as the sole source of information about diet and health. Students, parents, and even school officials were “afraid of” teachers in white coats, which gave the topic legitimacy, teachers said. One teacher paraphrased a parent’s perspective that “children listen more when it is someone in a white coat who speaks, it means the topic is important.” The cultural hierarchy did not simply represent the views of the public, but actively worked to challenge the autonomy of the teacher, even after the teacher had been trained. The pedagogical-skill orientation to nutrition knowledge was further reinforced by the subliminal message that nutrition knowledge is a part of clinical knowledge and not pedagogical skill. This is part of the reason that stakeholders would rather train a doctor than a nutritionist (62%, Figure 5), not necessarily as a reflection of the skills and qualifications of each, but as a symbol of authority. The institution and culture will continue to make the medical voice louder and louder, and no one, including the most knowledgeable teacher, will be an insider in nutrition education. This is where teacher training, no matter how well-intentioned, can go awry.

The third (and perhaps most systemic) barrier was the absence of state-made teaching modules that were relevant to the context. Despite the general understanding of the importance of FNE and the teacher’s desire to teach it purposefully, teachers were forced to rely on out-of-date pamphlets, generic handouts or *ad hoc* Internet resources, with none of this being contextualized to local food systems, seasonal foods or community dietary habits. We need hands-on training from trusted health care workers or dietitians, one teacher said, “We are not ready to teach food safety just from handouts.” Pedagogy was responsible for this difference, not content. There was a need for teachers to find ways to teach those facts in a way that would relate to their students’ lives, rather than just more facts. Theory, abstraction and disengagement were also seen in FNE, which was not fully realized in the context of the state’s failure to acknowledge that successful FNE depends not only on qualified teachers, but also on the development of the curriculum with their involvement, incorporation of the curriculum into their contexts and support from a system of resources. This absence was statistically significant (RQ1) as 79.4% of the teachers did not have formal training. Together, these three themes—lack of contextualized teaching materials for a diagnostic framework, cultural preference for medical authority and schedule compression—provide an explanation for why FNE remains a secondary objective and is not considered a core competency of Indian public schools. These themes also indicate an out-of-alignment system, as well as an inadequate resource base. Systems that actually hinder the delivery of FNE are in place when the policies are approved. These three interdependent barriers all need to be addressed simultaneously; if any intervention, no matter how well-intentioned, is not going to be superficial, as the data suggest. As the data suggest, if any intervention is not to be superficial, then all three of these interdependent barriers must be addressed simultaneously, through the securing of dedicated time, the redefinition of authority, and the co-development of context-specific curricula. The teachers in this study are not resistant to change; they are awaiting permission to teach what they know is important, not from parents or doctors, but from the system itself. The qualitative results are not only descriptive of barriers, but also provide a diagnostic architecture of systemic disempowerment. The three emergent themes of syllabus squeeze—the privileged cultural position of medical authority, and an absence of contextual relevance in the teaching modules—are not additive, isolated problems, but are mutually reinforcing mechanisms that combine to undermine teacher agency. Lack of state-designed, culturally-specific and pedagogically-scaffolded resources, as displayed in Table 2, has led to everyday food environments of children in rural villages and urban slums as a consequence of the Interlocking Systemic Barriers to Food and Nutrition Education.

**Table 2:** Three interlocking systemic barriers to food and nutrition education implementation: thematic synthesis from teacher and parent narratives.

Syllabus squeeze	Lack of dedicated time in the timetable; FNE treated as extracurricular	“It’s nearly impossible to squeeze FNE in when our syllabus is already full.” Primary Teacher
Cultural privileging of doctor-led authority	The perception that medical professionals hold greater legitimacy than teachers	“Children listen more when someone in a white coat speaks; it gives the topic importance.” Parent (cited by teacher)
Absence of state-mandated, contextually relevant modules	Lack of accessible, practical teaching resources aligned with local food systems	“We need training from actual nutritionists or doctors. Reading handouts alone does not equip us to teach food safety.” Government School Teacher

Column headers: Barrier Theme | Description | Representative Quote. Themes were derived from the inductive thematic analysis of 32 semi-structured interviews.

Each theme operates at a different level of the education system (structural, cultural, and institutional). Yet, they converge on one outcome: FNE is rendered a symbolic gesture rather than a pedagogical practice. The quantitative data confirm this diagnosis: teachers who report no training (RQ1) are precisely those who feel the least confident (RQ2), desire structural legitimacy the most (RQ3), and whose confidence is most strongly predicted by training exposure (RQ4). What emerges is not a list of problems but a coherent, interlocking system of neglect that can be dismantled only through coordinated, multi-level intervention. Table 3 synthesizes these findings into a policy-action framework (see Supplementary material). “As demonstrated in Table 3, the barriers to FNE implementation are not merely logistical; they are systemic, interdependent, and rooted in institutional misalignment.”

**Table 3:** Four research questions on teacher capacity in food and nutrition education: scoring framework and interpretive thresholds.

Research question (RQ)	Items	Scoring direction	Interpretive threshold	Rationale
RQ1: Training exposure	1.1, 1.2, 1.3	Reverse-scored	Mean score < 2.5 indicates inadequate training exposure	Higher scores reflect greater perceived inadequacy; aligns with 79.4% reporting no formal training
RQ2: Urban rural disparity in self-efficacy	2.1, 2.2, 2.3	Direct	Mean score ≥ 4.0 = high self-efficacy; urban vs. rural comparison	Reflects strong urban–rural gap (M_urban_= 3.5
RQ3: Correlation with curriculum preference	3.1, 3.2, 3.3	Direct	Mean score ≥ 4.0 = strong preference for standalone FNE	Supports significant correlation with self-efficacy (*r* = 0.73)
RQ4: Training as predictor of confidence	4.1, 4.2, 4.3	Direct	Mean score ≥ 4.0 = strong belief that training drives confidence	Validates training as strongest predictor (*β* = 0.54, *p* < 0.001)

All items were rated on a 5-point Likert scale: 1 = Strongly Disagree, 2 = Disagree, 3 = Neutral, 4 = Agree, and 5 = Strongly Agree.

## Discussion

The findings of the study all support the urgent conclusion that the main, yet frequently ignored, factors that either make global nutrition education mandates a reality or render them ineffective in India’s primary schools are public awareness, funding distribution, and teacher capacity policy rhetoric. The prevalence of 79.4% of teachers missing formal training (RQ1) is not merely a reflection of a lack of professional skills; rather, it is an institutional critique of a system that sees teachers as passive consumers of external content rather than as epistemic agents with the ability to influence the meaning of the curriculum. Because Duncombe et al. (11) showed in their co-design protocol for school-based HIIT interventions that fidelity, engagement, and sustainability increase dramatically when teachers are positioned as co-creators rather than implementers, this finding directly addresses the assumption that nutrition education could be delivered through top-down, health sector-led interventions, such as school feeding programs or one-time awareness drives. This assumption is common in many LMIC policy frameworks. This is supported by the study data: the study’s qualitative theme of “syllabus squeeze” reveals a systemic erasure of pedagogical agency rather than a lack of will; teachers are expected to deliver FNE without the necessary time, authority, or resources. Structural inequality posing as geographic variation is further revealed by the glaring urban–rural difference in self-efficacy (RQ2: *d* = 1.63). Urban teachers expressed significantly greater confidence in FNE delivery (RQ2: *M* = 3.50 vs. *M* = 2.03, *d* = 1.63), a finding supported qualitatively by interview accounts of easier access to district-level training networks, administrative support, and infrastructure. This is a result of institutional privilege rather than personal ability. Silva et al. (15) found that the lack of enabling conditions, such as time, materials, and institutional prioritization, was the reason why school-based interventions failed in resource-constrained rural settings, rather than teacher apathy. These findings are consistent with their cluster-randomized trial conducted in Brazil. However, they are made worse in the Indian context by the lack of a national FNE mandate in B.Ed. curricula, which leaves teachers to deal with this policy void. This stands in stark contrast to the mandatory Cooking and Nutrition curriculum in England, which incorporates teacher preparation into initial teacher education and is bolstered by nationally recognized resources (9). According to the data, systemic scaffolding, rather than individual motivation, is what drives teacher confidence in India, and without this scaffolding, even the best-laid plans fall apart.

The strong relationship between teacher self-efficacy and preference for a standalone FNE subject (RQ3: *r* = 0.73) reveals an important pedagogical insight: teachers are not requesting more content, but rather curricular legitimacy. Their interest in a particular subject is not logistical; rather, it is epistemological. This shows a rejection of FNE as an “add-on” to science or health and a desire for it to be recognized as a basic skill in foundational education. This result is in line with that of Weber et al. (7), who found that rather than their knowledge of nutrition science, biology teachers’ intention to teach sustainable nutrition was most strongly predicted by their opinion of the subject’s legitimacy within the curriculum. The study’s findings support this. FNE becomes invisible in the absence of timetabling, assessment, or accountability, and invisibility leads to disempowerment. This directly challenges the belief that incorporating FNE into already-existing topics is adequate, which is still maintained in some policy circles. Integration without institutionalization results in fragmentation rather than coherence, as Kupolati et al. (6) cautioned more than 10 years ago.

The regression model (RQ4) offers empirical support for reform since training exposure (*β* = 0.54) was the best predictor of implementation confidence, surpassing infrastructure, experience, and urban location. But it matters what kind of training that is. Teachers demand pedagogical support and reject theoretical training, according to the study’s qualitative data: “We need hands-on training from actual nutritionists or doctors, not just handouts.” This is in line with a quasi-experimental study by Fahlman et al. (8) that demonstrated that in-service training, along with useful instructional resources, not just content, provided notable increases in teacher self-efficacy and teaching intent. By showing how important it is to contextualize, co-develop, and integrate training into local food systems, this study goes one step further. Without this, training becomes performative compliance. This outcome stands in sharp contrast to the prevalent Poshan Abhiyaan model in India, which places more emphasis on growth tracking and micronutrient distribution than on developing pedagogical capacity. In their AFLY5 trial, Lawlor et al. (16) showed that dietary behaviour change was most significantly mediated by changes in parental support and teacher self-efficacy rather than direct instruction. This suggests that the influence of FNE happens through relational rather than informational channels.

Perhaps the biggest obstacle to successful implementation is a long-standing preference for medical authority over pedagogical knowledge. Consistent with the qualitative theme of cultural deference documented in this study, 62 % of survey respondents clearly preferred doctors as trainers, which is more symbolic confirmation of the institutional hierarchy than a logical evaluation of expertise. Despite their training, teachers are still viewed as non-experts; this perception may be reinforced by policy, the media, and parental expectations. This is consistent with the findings of Gwelo et al. in South Africa, where community deference to clinicians undermined school-based nutrition programs (25) and marginalized teachers, who are supposed to teach nutrition.

These results provide strong justifications for a paradigm change in the formulation of policy. The nebulous pledge to “experiential learning” in NEP 2020 is still insufficient. The formative role of education in maintaining behavioural change is completely ignored by the supplementation-based strategy of the Poshan Abhiyaan. Instead, a three-pronged institutional architecture is needed:

1. Every B.Ed. A course that includes England’s statutory Cooking and Nutrition framework (9) must include mandatory, credit-bearing FNE modules to guarantee that teachers arrive at the classroom with strengthened pedagogical competence rather than merely content knowledge.
2. Protected, ring-fenced time in the main schedule with official accountability procedures to demonstrate that FNE is essential to the right to a high-quality education (SDG 4);
3. Open-access, contextually grounded, state-developed educational modules that were co-designed with farmers, teachers, and local nutritionists. These modules ensure sustainability and relevance by utilizing seasonal produce, traditional food knowledge, and community practices. The framework of the study provides a replicable model for LMICs and has important implications for SDGs. It identifies training as the primary predictor (RQ4), tracks curriculum legitimacy (RQ3), assesses training exposure (RQ1), and identifies training as the core predictor (RQ4). When properly executed, FNE goes beyond merely granting access to education to meet SDG 4’s call for relevant and effective delivery. SDG 3-ensuring healthy lives, can be achieved by promoting lifelong food literacy that prevents diet-related illnesses. Children must be taught to value food systems rather than just calories if SDG 12 is to be achieved. SDG 10, the reduction of inequality, is directly addressed by empowering long-marginalized rural teachers as valid change agents. This study provides a diagnostic and prescriptive model for global FNE reform in addition to documenting obstacles. This study has some limitations. The sample may not be representative of private schools or other states in India. The five rural schools were located in a relatively close geographical area to Chennai, meaning that there may be less generalisability to more distant settings. The cross-sectional study design does not allow for causal inference, and self-reported survey data can have social desirability bias. Another limitation of the study is that the frequency of teachers’ use of FNE was not systematically recorded at present. Longitudinal studies with larger geographic sampling of the population should be conducted to evaluate the scalability of this framework for future research. This study does not offer a localized fix, but a diagnostic framework: measure training exposure, map self-efficacy disparities, assess curricular legitimacy, and identify training as the core predictor of change. Wherever teachers are expected to deliver nutrition education without being entrusted as its architects, the same cycle of disempowerment repeats. The solution lies not in more pamphlets, more assemblies, or more external experts, but in redefining the teacher’s role: from passive transmitter to recognized expert, from implementer to curriculum co-designer. Only then will food and nutrition education move from the periphery of policy documents to the core of pedagogical practice.

## Conclusion

This study provides empirical evidence that the lack of embedding of food and nutrition education in the government primary schools of Tamil Nadu is not incidental nor inevitable, but is a result of identifiable, addressable institutional deficits. The results of the four research questions all lead to a clear and unmistakable diagnosis: the capacity of teachers, the combination of formal education, pedagogical confidence, curricular legitimacy, and institutional support, is the key factor in the realization or symbolization of global nutrition education mandates. It is not a statement of “just a number” that 79.4% of teachers received no formal nutrition education training since they are not the policy’s architect, but rather a passive recipient of the policy. The statistically significant urban–rural difference in self-efficacy (*d* = 1.63) also indicates that the gap in self-efficacy is not evenly distributed but is particularly large for the most resource-poor teachers serving the most nutritionally vulnerable children. The high level of correlation between teacher confidence and teacher preference for an FNE subject as a standalone course (*r* = 0.73) throws into question the reform agenda, and what teachers want is not more content but more curricular legitimacy and structural recognition. Training exposure was the strongest predictor of implementation confidence (*β* = 0.54), outpacing experience, infrastructure, and location, making clear a policy priority for investment based on evidence. In India and other low- and middle-income countries, the message is clear: sustainable food and nutrition education will need to be built into pre-service teacher education as compulsory credit-bearing courses, have their timetables ring-fenced, and be co-developed, with context. The gap between the policy and the classroom will remain until teachers are seen not as a source of nutrition information but as a valid pedagogical authority in food literacy.

## Data Availability

The original contributions presented in the study are included in the article/Supplementary material, further inquiries can be directed to the corresponding author.
