# Supplementary material for: From policy to practice: teacher capacity as the missing link in food and nutrition education reform in Tamil Nadu, India
Source: Front Nutr. 2026 Jul 1;13:1743897. doi: 10.3389/fnut.2026.1743897 (PMC13368667; doi:10.3389/fnut.2026.1743897)
Supplement: Supplementary file 1 [file Table_1.pdf]

ANNEXURE

FOOD AND NUTRITION EDUCATION (FNE) TEACHER CAPACITY SURVEY

SECTION A: DEMOGRAPHIC INFORMATION

(Please tick ONE option per row)

Target Respondents: Primary School Teachers (Grades 15), Government Schools, Tamil Nadu

Please indicate your level of agreement with each statement below by ticking only one box per item. There are no right or wrong answers. Your responses are anonymous and will be used solely for research purposes.

Section-A Demographic Table

Demographic Variable	Option 1	Option 2	Option 3	Option 4	Option 5
Gender	☐ Male	☐ Female	☐ Other	☐ Prefer not to say	
School Location	☐ Urban	☐ Rural	☐ Semi-Urban		
Years of Teaching Experience	☐ <2 years	☐ >2 years	☐ <6 years	☐ <15 years	☐ >15 years
Highest Educational Qualification	☐ 10th grade or below	☐ 12th grade / Diploma	☐ Undergraduate (BA/BSc/Bed)	☐ Postgraduate (MA/MSc/Med)	☐ Doctorate or equivalent
Primary Subject Taught	☐ All subjects (generalist)	☐ Language (Tamil/English)	☐ Mathematics	☐ Environmental Science	☐ Other: ________

Section B. Teacher Capacity Survey Instrument:

RQ	Theme	Item Statement	1	2	3	4	5
RQ1: Training Exposure	Formal Training Received	I have received structured, credit-bearing training on nutrition pedagogy from a recognized institution.	☐	☐	☐	☐	☐
		I have been trained in the science of food processing, nutritional requirements, and safe handling for children.	☐	☐	☐	☐	☐
		My training in nutrition education was practical, not just theoretical.	☐	☐	☐	☐	☐
RQ2: UrbanRural Disparity in Self-Efficacy	Confidence in Delivery	I feel confident teaching students about balanced diets and healthy food choices.	☐	☐	☐	☐	☐
		I can adapt FNE content to suit the local food culture and resources of my students.	☐	☐	☐	☐	☐
		I feel adequately prepared to respond to students 'questions about nutrition and food sustainability.	☐	☐	☐	☐	☐

RQ3: Correlation with Curriculum Preference	Curriculum Legitimacy	I believe FNE should be taught as a standalone subject, not embedded in science or social studies.	☐	☐	☐	☐	☐
		If FNE were a standalone subject, I would be more motivated to plan and deliver it effectively.	☐	☐	☐	☐	☐
		Having FNE as a formal subject would signal to the school system that it is a priority, not an add-on.	☐	☐	☐	☐	☐
RQ4: Training as Predictor of Confidence	Training → Confidence Link	My confidence in teaching FNE is directly tied to whether I have received professional training in it.	☐	☐	☐	☐	☐
		Even if I have good intentions, I cannot teach FNE well without formal training in pedagogical methods.	☐	☐	☐	☐	☐
		Training in nutrition pedagogy matters more than just knowing nutritional facts; it changes how I teach.	☐	☐	☐	☐	☐

12-item 5-point Likert Scale Measuring FNE Implementation Readiness in Tamil Nadu's Government Primary Schools.

Semi-structured Interviews

Section A: Demographic Profile

Variable	Response Options
Gender	☐ Male ☐ Female ☐ Other ☐ Prefer not to say
School Location	☐ Urban ☐ Rural

Years of Teaching Experience	☐ <2 years ☐ 25 years ☐ 610 years ☐ 1115 years ☐ >15 years
Highest Educational Qualification	☐ 10th grade or below ☐ 12th grade / Diploma ☐ Undergraduate (B.A./B.Sc./B.Ed.) ☐ Postgraduate (M.A./M.Sc./M.Ed.) ☐ Doctorate or equivalent
Primary Subject Taught	☐ All subjects (generalist) ☐ Language (Tamil/English) ☐ Mathematics ☐ Environmental Science ☐ Other: ________

Section B: Semi-structured Interview

RQ1 Training Exposure	What kind of training, if any, have you received to teach food and nutrition topics to your students?	Who provided the training? Was it sufficient? What areas were most useful or missing?
RQ2 Preference for Standalone Subject	Would you prefer food and nutrition education to be taught as a separate subject or integrated within existing subjects? Why?	What advantages or challenges do you see in each approach? How would it impact your workload?
RQ3 Self-Efficacy in FNE Delivery	When you tried to teach nutrition in class, what made you feel it worked and what made you feel it didn't?	Can you share a specific example? How confident do you feel when handling student questions?
RQ4 Perceived Institutional Support	What forms of support (materials, time, encouragement) have you received from your school or administration for teaching nutrition?	What additional support would make your job easier? How does the current timetable affect nutrition teaching?
